# LncRNA HCG18 promotes osteosarcoma growth by enhanced aerobic glycolysis via the miR-365a-3p/PGK1 axis

**DOI:** 10.1186/s11658-021-00304-6

**Published:** 2022-01-06

**Authors:** Xiaohui Pan, Jin Guo, Canjun Liu, Zhanpeng Pan, Zhicheng Yang, Xiang Yao, Jishan Yuan

**Affiliations:** 1 Department of Orthopedics, The Affiliated People’s Hospital of Jiangsu University, Zhenjiang, 212002 Jiangsu China; 2 Department of Orthopedics, Changzhou No. 2 People’s Hospital, The Affiliated Hospital of Nanjing Medical University, Changzhou, China; 3 Department of Orthopedics, Zhenjiang First People’s Hospital Branch, Zhenjiang, People’s Republic of China; 4 Department of Respiratory Therapy, The Affiliated People’s Hospital of Jiangsu University, Zhenjiang, 212002 Jiangsu China

**Keywords:** HCG18, miR-365a-3p, PGK1, Osteosarcoma, Aerobic glycolysis

## Abstract

**Background:**

Osteosarcoma (OS) is a common primary bone malignancy. Long noncoding RNA HCG18 is known to play an important role in a variety of cancers. However, its role in OS and relevant molecular mechanisms are unclear.

**Methods:**

Real-time quantitative PCR was performed to determine the expression of target genes. Function experiments showed the effects of HCG18 and miR-365a-3p on OS cell growth.

**Results:**

HCG18 expression was increased in OS cell lines. Moreover, in vitro and in vivo experiments demonstrated that HCG18 knockdown inhibited OS cell proliferation. Mechanistically, HCG18 was defined as a competing endogenous RNA by sponging miR-365a-3p, thus elevating phosphoglycerate kinase 1 (PGK1) expression by directly targeting its 3ʹUTR to increase aerobic glycolysis.

**Conclusion:**

HCG18 promoted OS cell proliferation via enhancing aerobic glycolysis by regulating the miR-365a-3p/PGK1 axis. Therefore, HCG18 may be a potential target for OS treatment.

**Supplementary Information:**

The online version contains supplementary material available at 10.1186/s11658-021-00304-6.

## Background

Osteosarcoma (OS) is a common primary bone tumor and originates from interstitial tissue [1]. It accounts for approximately 3%-5% of malignant tumors and is particularly common in those aged under 20 years [2]. With the development of OS treatment including surgery combined with neoadjuvant radiotherapy and chemotherapy, the 5-year survival rate of OS has increased to 60% [3]. Nevertheless, the overall OS survival rate has not greatly improved in the last few decades because of the unclear molecular mechanisms of OS. Consequently, it is essential to investigate the molecular mechanisms underlying OS development and to find new therapeutic targets for its treatment.

Only 2% of the RNA in the human genome can be translated into protein, the remaining RNA being non-coding RNA (ncRNA) [4, 5]. Among ncRNAs, long noncoding RNAs (lncRNAs) are transcripts of greater than 200 bp, with limited or no protein coding ability [6]. Studies have shown that lncRNAs participate in many cellular activities, including gene translation and transcription and protein synthesis [7–10]. In addition, lncRNA serves as a competing endogenous RNA (ceRNA) to influence the expression of messenger RNA (mRNA) by interacting with microRNAs (miRNAs) [11]. It is reported that lncRNAs act as an essential factor in OS occurrence and development [12]. The lncRNA HCG18 was initially identified as a ceRNA that promotes intervertebral disc degeneration via the miR-146a-5p/TRAF6 axis [13]. Accumulating evidence indicated that HCG18 is an oncogene in numerous tumors, including gastric cancer, clear cell renal cell carcinoma, and hepatocellular carcinoma [14–16]. However, the role and function of HCG18 in OS remain rarely reported.

It has been reported that miRNAs frequently act as tumor suppressors in different human cancers [17–19]. LncRNAs can sponge miRNAs to reduce the suppressive effect of miRNAs in OS. For example, lncRNA RUSC1-AS1 was shown to regulate OS development through inhibiting miR-101-3p, which increased Notch1 expression [20]. LncRNA H19 expression was increased in OS and promoted OS cell proliferation and invasion by regulating the miR‑29a‑3p/LASP1 axis [21]. MiR-365a-3p has been reported to act as an anti-oncogene and mediates TET1 suppression of Hep-2 cell growth [22]. However, the function of miR-365a-3p in OS has not been reported.

Cancer cells obtain material and energy to meet their rapid growth through aerobic glycolysis, which is termed the Warburg effect [23, 24]. Studies have shown that various metabolic enzymes and signaling molecules that are involved in glucose metabolism display an essential role in tumor genesis and development [25]. These metabolic enzymes and signaling molecules are considered as an important target for diagnosis and treatment of malignant tumors. Phosphoglycerate kinase 1 (PGK1) is a key enzyme of glycolysis. Moreover, it has been reported to be a cancer promoter, having significant effects on various forms of cancers, including lung cancer, pancreatic cancer, breast cancer, and liver cancer [26–29]. Previous studies have demonstrated that PGK1 expression was greatly upregulated in cisplatin-resistant OS tissues [30]. However, the function of PGK1 in OS growth and its corresponding molecular mechanisms are unknown.

In this study, we demonstrated that lncRNA HCG18 was upregulated in OS cell lines and elevated OS proliferation by increasing aerobic glycolysis. Mechanistically, HCG18 competed for miR-365a-3p with PGK1, which is a key glycolytic coding mRNA, and moderated the repressive effect of miR-365a-3p on PGK1, thereby resulting in increased PGK1 expression and aerobic glycolysis. Therefore, the HCG18/miR-365a-3p/PGK1 axis might be a new therapeutic target in the treatment of OS.

## Materials and methods

### Cell culture and reagents

The Cell Bank of the Chinese Academy of Sciences (Shanghai, China) provided the human osteoblast hFOB1.19 (catalog number: GNHu14) cell line and the MG63 (catalog number: TCHu124) and MNNG-HOS (catalog number: TCHu167) human OS cell lines. The U-2OS (catalog number: HTB-96) and 143B (catalog number: CRL-8303) OS cell lines were purchased from the American Type Culture Collection (ATCC, Manassas, VA, USA). Briefly, the human OS cell lines (MG63, MNNG-HOS, U-2OS, and 143B cells) were maintained at 37 °C under a 5% CO_2_ atmosphere, while hFOB1.19 cells were maintained at 34.5 °C under a 5% CO_2_ atmosphere. The antibodies used in this study were against PGK1 (ab199438; Abcam, Cambridge, UK) and GAPDH (ab8245; Abcam, Cambridge, UK).

### RNA transfection

Lipofectamine 3000 (Invitrogen, Carlsbad, CA, USA) was used according to the manufacturer's protocol. The miR-365a-3p mimic, control inhibitor, miR-365a-3p inhibitor, inhibitor NC, pcDNA-PGK1 and pcDNA-NC were all obtained from Gene-Pharma (Shanghai, China). Lentiviral infection was employed to generate stably transfected OS cell lines. The plasmid for HCG18 knockdown (sh-HCG18) and the empty plasmid (sh-CON) were obtained from Gene-Pharma (Shanghai, China). To establish stable HCG18-knockdown cell lines, the target cells were co-infected with 1 × 10^8^ lentivirus transducers and polybrene (Sigma-Aldrich, St. Louis, MO, USA). Furthermore, 2.5 μg/mL of puromycin was used to screen the infected cells after 72 h.

### Real-time quantitative PCR (RT-qPCR)

Total RNA from OS cells and tissues was extracted using TRIzol (Invitrogen) according to the manufacturer’s protocol. RT-qPCR was performed as we reported previously [31]. The relative mRNA expression was calculated with the 2 − ∆∆Ct method. GAPDH was used as the internal control. The primers used were as follows: HCG18 sense: 5′-ATCCTGCCAATAGATGCTGCTCAC-3ʹ; anti-sense: 5′-AGCCACCTTGGTCTCCA GTCTC-3′; GAPDH sense: 5′-CCAGCAAGAGCACAAGAGGAAGAG-3ʹ; anti-sense: 5′-GGTCTACATGGCAACTGTGAGGAG-3′; miR-365a-3p sense: 5′-TAAT GCCCCTAAAA ATCCTTAT-3′, anti-sense: 5′-CAGTGCGTGTCGTGGAGT-3′; PGK1 sense: 5′-TTCTGTTCTTGAAGGACTGTGT-3′; anti-sense: 5′-CTTTAACC TTGTTCCCAGAA GC-3′.

### Western blotting

Western blotting was performed as previously described [31]. Briefly, total protein was lysed with RIPA buffer (Sigma, USA). Subsequently, protein samples were electrophoresed on 12% SDS-PAGE gels and transferred to a membrane. The membrane was incubated with primary antibodies overnight at 4 °C. The membrane was subsequently incubated with secondary antibody for 1 h at room temperature and detected by enhanced chemiluminescence.

### Cell counting kit (CCK)-8 assay

Firstly, differently treated 3 × 10^3^ OS cells were seeded into 96-well plates. Following culture for 0, 24, 48, or 72 h under a 5% CO_2_ atmosphere at 37 °C, 10 μL of CCK-8 solution (C6005, New Cell & Molecular Biotech, Shanghai, China) was added to every well, and the OS cells were incubated for 2 h. The absorbance at 450 nm was determined using a microplate reader (Bio-Rad, USA).

### Colony formation assay

A total of 1 × 10^3^ differently treated OS cells were seeded into six-well plates and cultured for 10 days. The cells were fixed with paraformaldehyde and dyed with 0.5% (w/v) crystal violet. Finally, the cell colonies were counted.

### EdU assay and cell apoptosis assay

The EdU kit (Beyotime, shanghai, China) and cell apoptosis kit (share-bio, shanghai, China) were applied to measure cell growth as previously described [32].

### Dual-luciferase reporter assay

The dual-luciferase reporter assay was carried out to evaluate the relationship among HCG18, miR-365a-3p, and PGK1 according to the instructions as we previously described [32].

### RNA immunoprecipitation (RIP) assay

The relationship between HCG18 and miR-365a-3p was evaluated by RIP assays. The experiment was measured following the manufacturer's instructions.

### Mouse xenograft assay

The animal study was carried out according to the guidelines of the Research Ethics Committee of East China Normal University. For the in vivo metastasis model, ten male BALB/C nude mice (5–6 weeks old) were randomly divided into two groups (vector and sh-HCG18 groups) and subcutaneously injected with 1.5 × 10^6^ cells. The volume and weight of the tumors were determined every 5 days. The mice were euthanized after 20 days. The subcutaneous tumors were collected and measured. All animal protocols were approved by the Affiliated Hospital of Nanjing Medical University Animal Protection and Use Committee.

### Measurement of oxidative phosphorylation and glycolysis

The oxygen consumption rate (OCR) and extracellular acidification rate (ECAR) of different transfected OS cells were measured through an XF96 metabolic flux analyzer (Seahorse Biosciences, Billerica, MA, USA) according to the manufacturer’s protocol.

### Measurement of cellular ATP level and lactate production

The cellular ATP level and lactate production were determined as previously described [32]. An ATP assay kit (Promega, Madison, WI) was used to measure the cellular ATP level and a lactate assay kit (BioVision, USA) was used to detect extracellular lactate levels.

### TUNEL assay and immunohistochemical staining

The apoptosis of transfected OS cells in the xenograft tumors was detected using the TUNEL kit (Roche, Basel, Switzerland), as we previously described. Ki67 was measured using a primary antibody (1:200, GB13030; Servicebio, Wuhan, China).

### Statistical analysis

Statistical analyses were performed using GraphPad Prism 8.0 and SPSS 26.0. Data were presented as mean ± standard deviation. After testing the homogeneity of variance, two-tailed Student’s t-test was applied to compare the results from different groups. All experiments were performed in triplicate. P < 0.05 was considered statistically significant.

## Results

### HCG18 knockdown inhibited OS proliferation in vitro

**Fig. 1:**
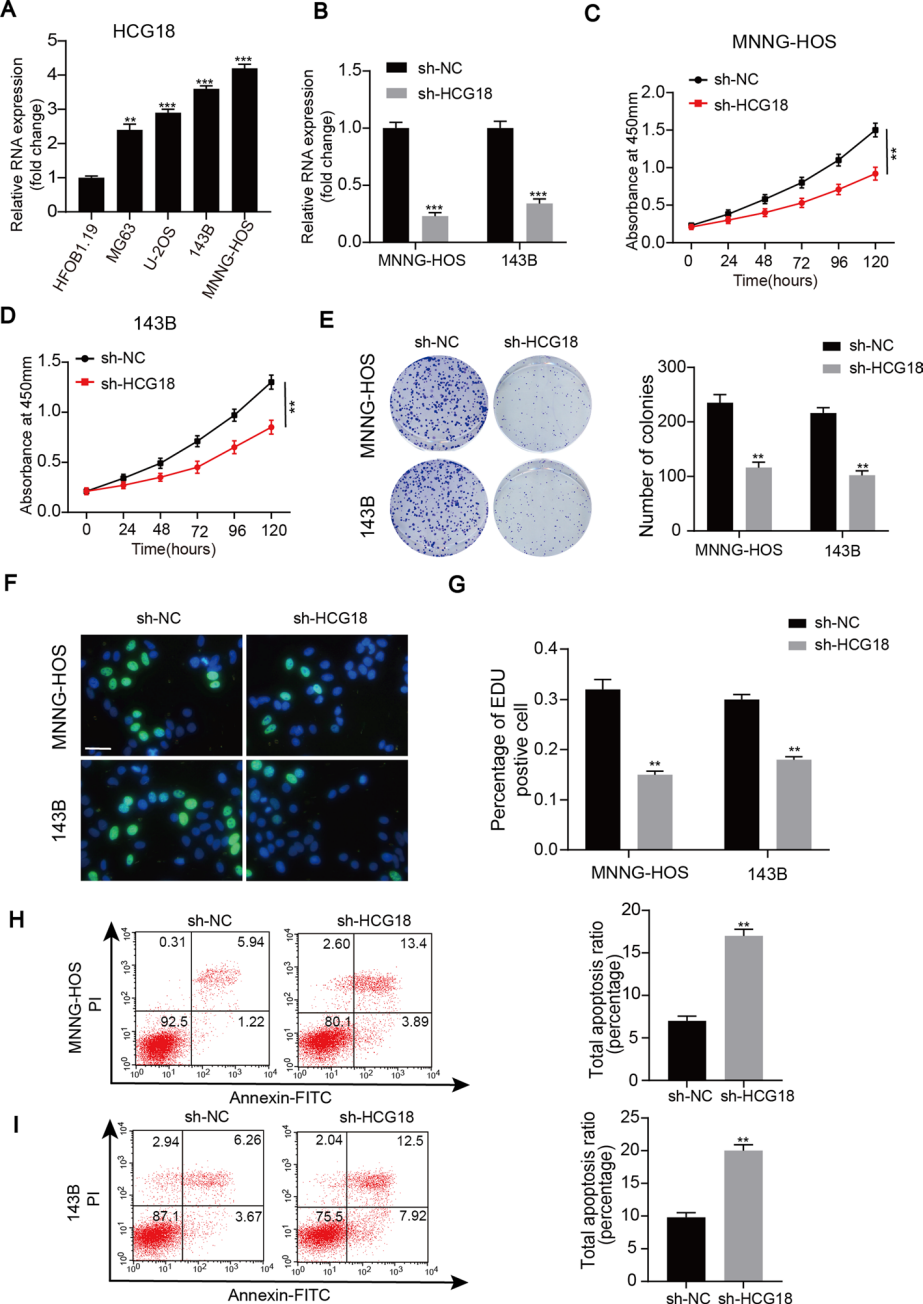
HCG18 knockdown inhibited OS proliferation in vitro.** A** HCG18 expression in OS cell lines and in hFOB1.19. **B** Knockdown efficiency of HCG18 in OS cells. CCK8 (**C** and **D)**, colony formation (**E**), EDU (**F** and **G**) assays performed with OS cells transfected with sh-HCG18 or sh-CON. **H** and **I** Cell apoptosis was determined in the OS cells by flow cytometry after transfection with sh-HCG18. Knockdown of HCG18 significantly induces apoptosis of OS cells. ** represents *P* < 0.01, *** represents *P* < 0.001. All experiments were repeated three times

To determine whether lncRNA HCG18 plays a critical role in OS progression, we first detected the expression of HCG18 in human OS cell lines (MG63, MNNG-HOS, U-2OS, and 143B) and a human osteoblast cell line (hFOB1.19). HCG18 expression was upregulated in all the OS cell lines, particularly in the MNNG-HOS and 143B cell lines (Fig. 1A). Figure 1B shows the knockdown efficiency of HCG18 in these two OS cell lines (MNNG-HOS and 143B). As displayed in Fig. 1C–G, HCG18 knockdown suppressed the cell proliferation of OS cells through the CCK-8, colony formation assay and EdU assay. Additionally, we applied flow cytometry to determine that, compared with the control cells, knockdown of HCG18 remarkably increased the apoptosis of the OS cells (Fig. 1H and I). These data indicated that HCG18 was upregulated in the OS cell lines and its knockdown inhibited OS cell proliferation in vitro.

### HCG18 enhanced aerobic glycolysis in OS cells

**Fig. 2:**
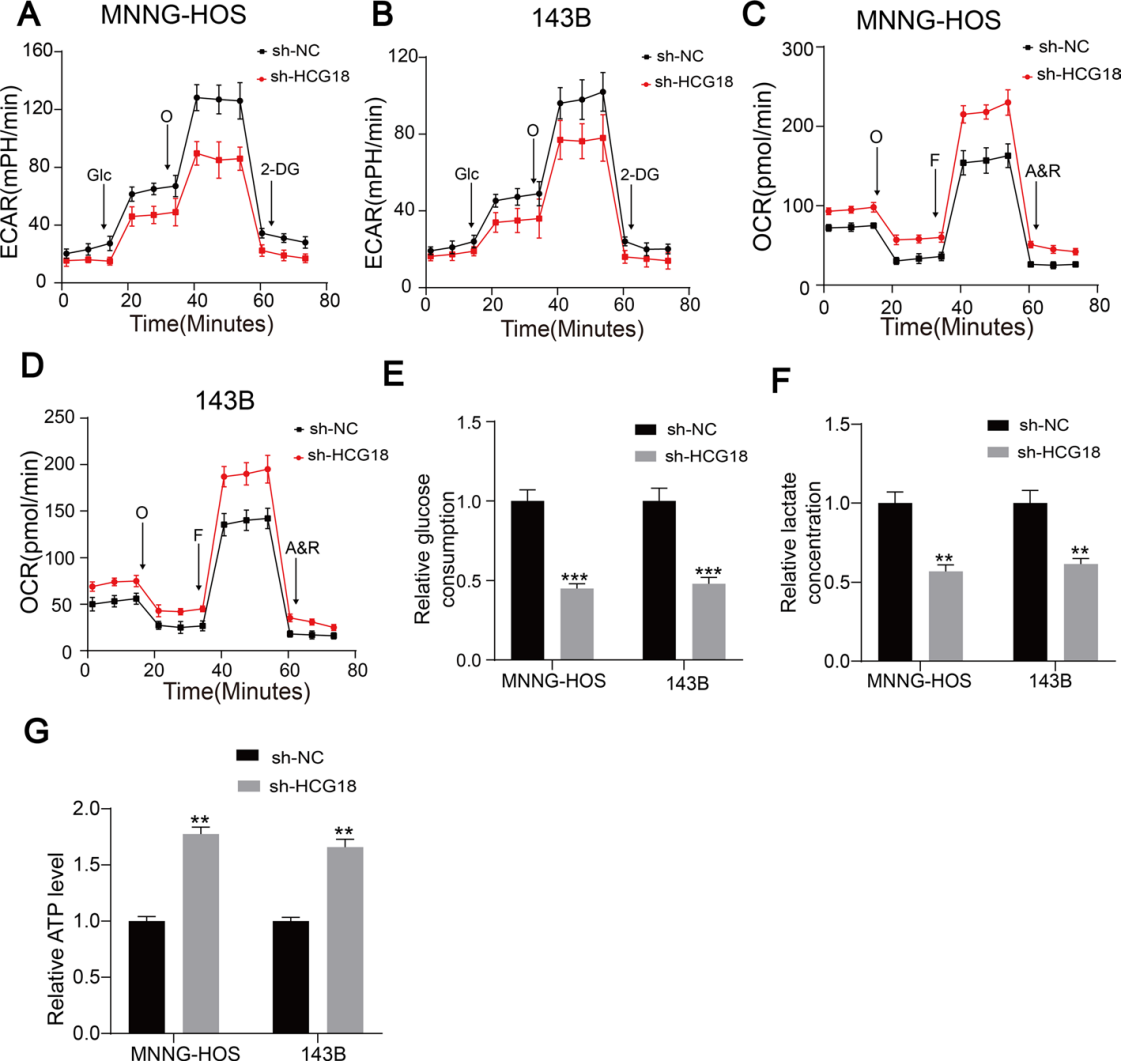
HCG18 enhanced aerobic glycolysis in OS cells.** A** and **B** ECAR of sh-Control and sh-HCG18 group was detected. Glc: glucose, Oligo: oligomycin, 2-DG: 2-deoxy-d-glucose. **C** and **D** OCR of sh-Control and sh-HCG18 group was measured. O: Oligomycin, F: FCCP, A&R: antimycin A/rotenone. Glucose consumption (**E**), lactate production (**F**), and ATP level (**G**) demonstrated the effect of HCG18 knockdown on aerobic glycolysis in OS cells. ** represents *P* < 0.01, *** represents *P* < 0.001. All experiments were repeated three times

In the presence of oxygen, cancer cells display a higher rate of glucose consumption and lactate production than their normally differentiated counterparts [33]. Further evidence showed that aerobic glycolysis played an important role in OS progression [34, 35]. Interestingly, Fig. 2A–D shows that HCG18 knockdown partly reduced the ECAR in MNNG-HOS and 143B cells but enhanced the OCR of these cells. Moreover, HCG18 knockdown reduced glucose consumption in OS cells (Fig. 2E). As depicted in Fig. 2F and G, the ATP level increased and the production of lactate decreased in the HCG18-knockdown OS cells. Collectively, these results indicated that HCG18 knockdown inhibited aerobic glycolysis in OS cells.

### PGK1 was the target of HCG18

**Fig. 3:**
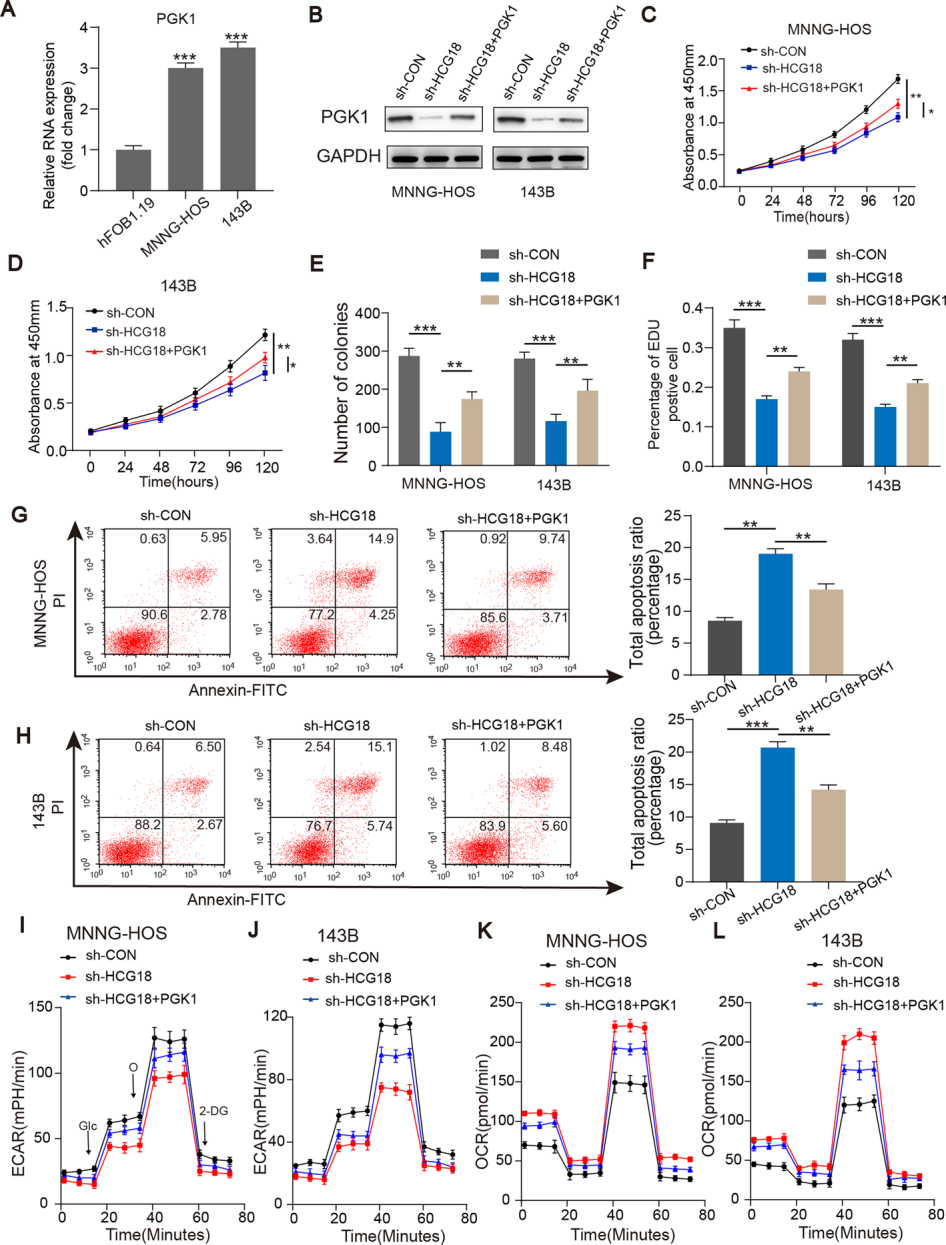
PGK1 was the target of HCG18.** A** PGK1 expression in OS cell lines and in hFOB1.19. **B** Overexpression efficacy of PGK1 in sh-HCG18 OS cells. CCK8 (**C** and **D**), colony formation (**E**), EDU (**F**), and apoptosis (**G** and **H**) assays confirmed that overexpression of PGK1 partly reversed the suppressive effects of HCG18 knockdown on OS growth. **I-L**, ECR and OCR in OS cells in different groups (sh-Control, sh-HCG18, and sh-HCG18 + ov-PGK1) were measured. * represents *P* < 0.05, ** represents *P* < 0.01, *** represents *P* < 0.001. All experiments were repeated three times

To investigate how HCG18 regulates glycolysis in OS, the levels of various genes involved in the glycolytic pathway and tricarboxylic acid (or Krebs) cycle were detected by qRT-PCR in HCG18 knockdown MNNG-HOS and 143B cells. The data indicated that HCG18 knockdown greatly reduced mRNA expression of PGK1, a key glycolytic gene, in OS cells (Additional file 1: Fig. S1A and B). We also found that PGK1 expression was increased in the OS cell lines (Fig. 3A). Subsequently, we overexpressed PGK1 in wild-type (WT) and HCG18 knockdown OS cell lines and measured the overexpression efficiency (Fig. 3B). As depicted in Fig. 3C–F, overexpression of PGK1 enhanced cell proliferation of sh-HCG18-transfected OS cells. The cell apoptosis assay indicated that overexpression of PGK1 reduced the apoptosis rate of sh-HCG18-transfected OS cells (Fig. 3G and H). Additionally, the Warburg effect in the OS cell lines was improved by overexpressing PGK1 (Fig. 3I–L). Overall, these results confirmed that HCG18 regulated PGK1-mediated glycolysis and thereby improved OS proliferation.

### MiR-365a-3p targeted PGK1 directly in OS cells and inhibited OS growth

**Fig. 4:**
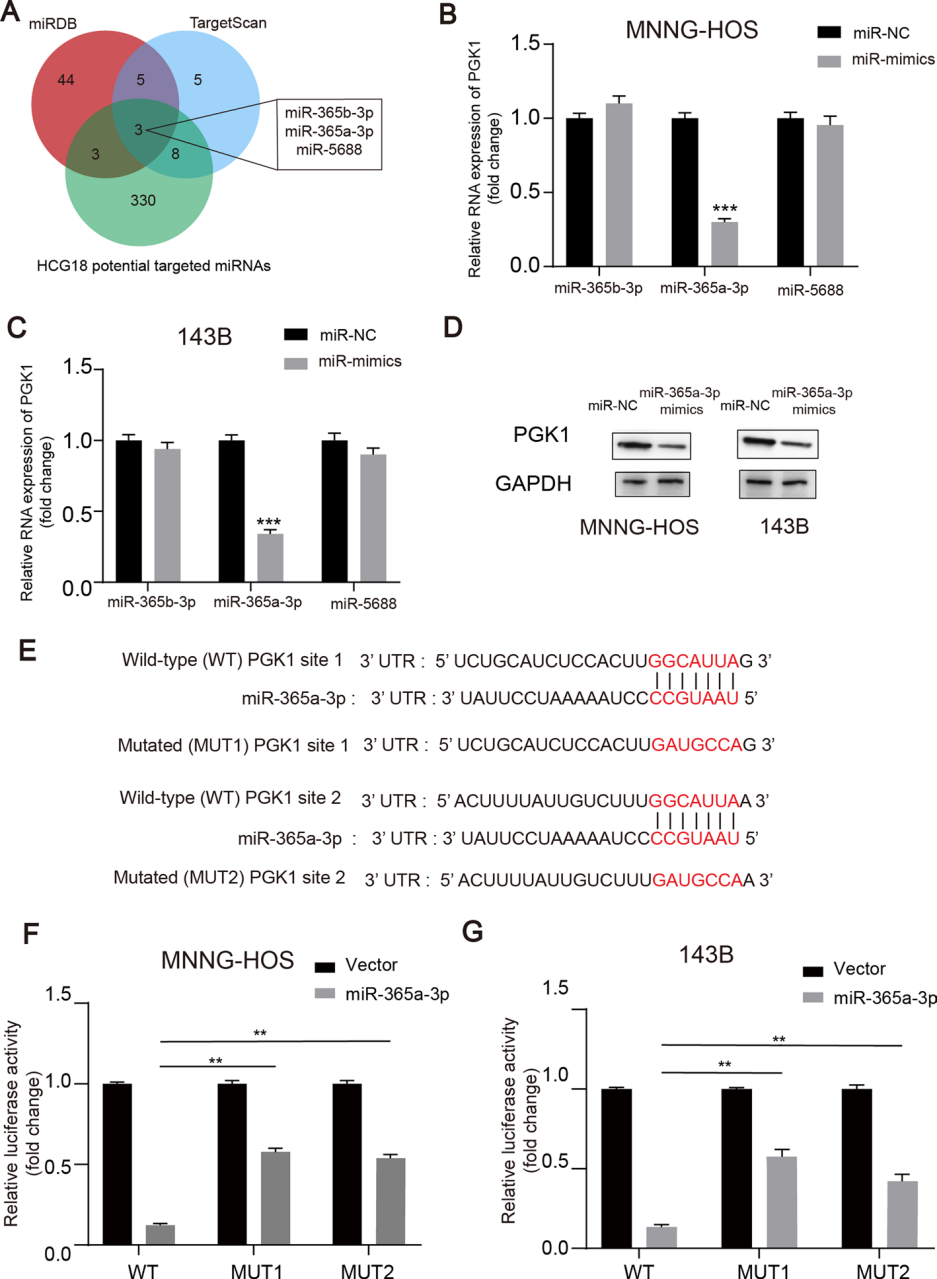
MiR-365a-3p targeted PGK1 directly in OS cells.** A** Venn diagram showing the predicted target genes of PGK1 and HCG18 from databases (miRDB, TargetScan, and StarBase). **B** and **C** PGK1 expression in miRNA mimics the treated or Control MNNG-HOS and 143B cells. **D** PGK1 expression was measured by western blot in OS cells transfected with miR-365a-3p mimics or negative control. **E** Putative binding sites between PGK1 and miR-365a-3p. **F** and **G** Dual-luciferase reporter assay in OS cells transfected with miR-365a-3p mimics or negative control. ** represents *P* < 0.01, *** represents *P* < 0.001. All experiments were repeated thrice

It has been reported that various lncRNAs act as ceRNAs and sponge miRNAs to regulate the expression of target genes [36, 37]. We hypothesized that HCG18 also regulates miRNAs in the form of a sponge molecule to regulate PGK1 in OS. First, we used prediction algorithms, including TargetScan, miRDB, and StarBase, and identified three potential miRNAs that are regulated by HCG18 and that target PGK1 (Fig. 4A). Subsequently, we overexpressed these miRNAs in OS cells and when applying RT-qPCR found that the expression of PGK1 was reduced in the OS cells with miR-365a-3p overexpression (Fig. 4B and C). Furthermore, western blotting was performed to confirm that miR-365a-3p overexpression reduced protein expression of PGK1 in the OS cells (Fig. 4D). The potential target site of miR-365a-3p on PGK1 is shown in Fig. 4E. Subsequently, we performed dual luciferase reporter assays to investigate the relationship between miR-365a-3p and PGK1. As shown in Fig. 4F and G, the miR-365a-3p mimic decreased the luciferase activity of the WT PGK1 3′UTR reporter, but not in the mutant 3′UTR of the PGK1 reporter in OS cells. Collectively, these data showed that PGK1 is a target of miR-365a-3p in OS cells.

It was reported that miR-365a-3p acts as a cancer suppressor gene in various cancers [22, 38, 39]. Therefore, we investigated whether miR-365a-3p inhibits OS growth. Firstly, miR-365a-3p was present at a higher level in the OS cell lines compared to the HFOB1.19 cell line (Additional file 2: Fig. S2A). We overexpressed miR-365a-3p in the MNNG-HOS and 143B cell lines and the efficiency was determined by RT-qPCR (Additional file 2: Fig. S2B). Through colony formation, EdU, and the cell apoptosis assay, we found that overexpression of miR-365a-3p inhibited OS proliferation and increased apoptosis of these OS cells (Additional file 2: Fig. S2D–G).

### HCG18 acted as a molecular sponge of miR-365a-3p

**Fig. 5:**
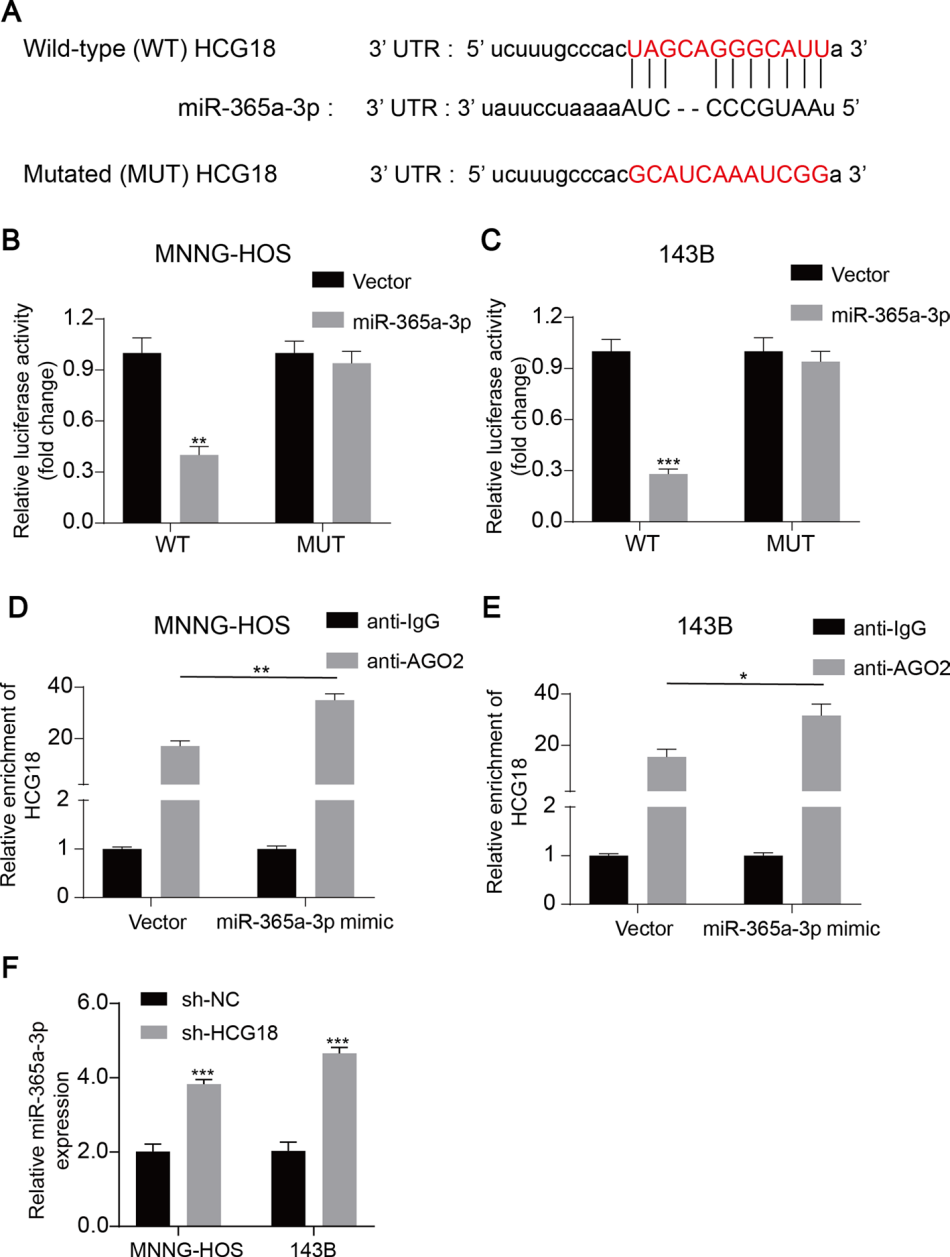
HCG18 acted as a molecular sponge of miR-365a-3p.** A** Putative binding sites between HCG18 and miR-365a-3p. **B** and **C** Luciferase activity of OS cells in luciferase reporter plasmid containing wild-type HCG18 3ʹ-UTR and mutant HCG18 3ʹ-UTR co-transfected with miR-365a-3p mimics or negative control was assessed. **D** and **E**, AGO2-RIP followed by qPCR to detect HCG18 level after miR-365a-3p overexpression. F, Expression of miR-365a-3p was upregulated in OS cells transfected with HCG18 shRNA or the control shRNA by RT-qPCR. * represents *P* < 0.05, ** represents *P* < 0.01, *** represents *P* < 0.001. All experiments were repeated three times

To further determine whether HCG18 regulated gene expression by functioning as a sponge of miR-365a-3p in OS cells, we used StarBase analysis and revealed that HCG18 had a putative binding site on miR-365a-3p (Fig. 5A). Dual-luciferase reporter assay was carried out and the results showed that overexpression of miR-365a-3p inhibited the luciferase activity of HCG18-WT but not HCG18-MUT (Fig. 5B and C). Ago2 is a crucial factor of the RNA-induced silencing complex [40]. Anti-Ago2 RIP was conducted in miR-365a-3p-transfected OS cells transfected with miR-365a-3p. The enrichment of endogenous HCG18 was improved after miR-365a-3p overexpression in OS cells (Fig. 5D and E). Subsequently, we detected the miR-365a-3p expression in HCG18-knockdown and control OS cells. Knockdown of HCG18 significantly increased miR-365a-3p expression in OS cells (Fig. 5F). Taken together, these results showed that HCG18 functioned as a sponge for miR-365a-3p in OS cells.

### MiR-365a-3p knockdown reversed the suppressive effect induced by HCG18 knockdown in OS cells

**Fig. 6:**
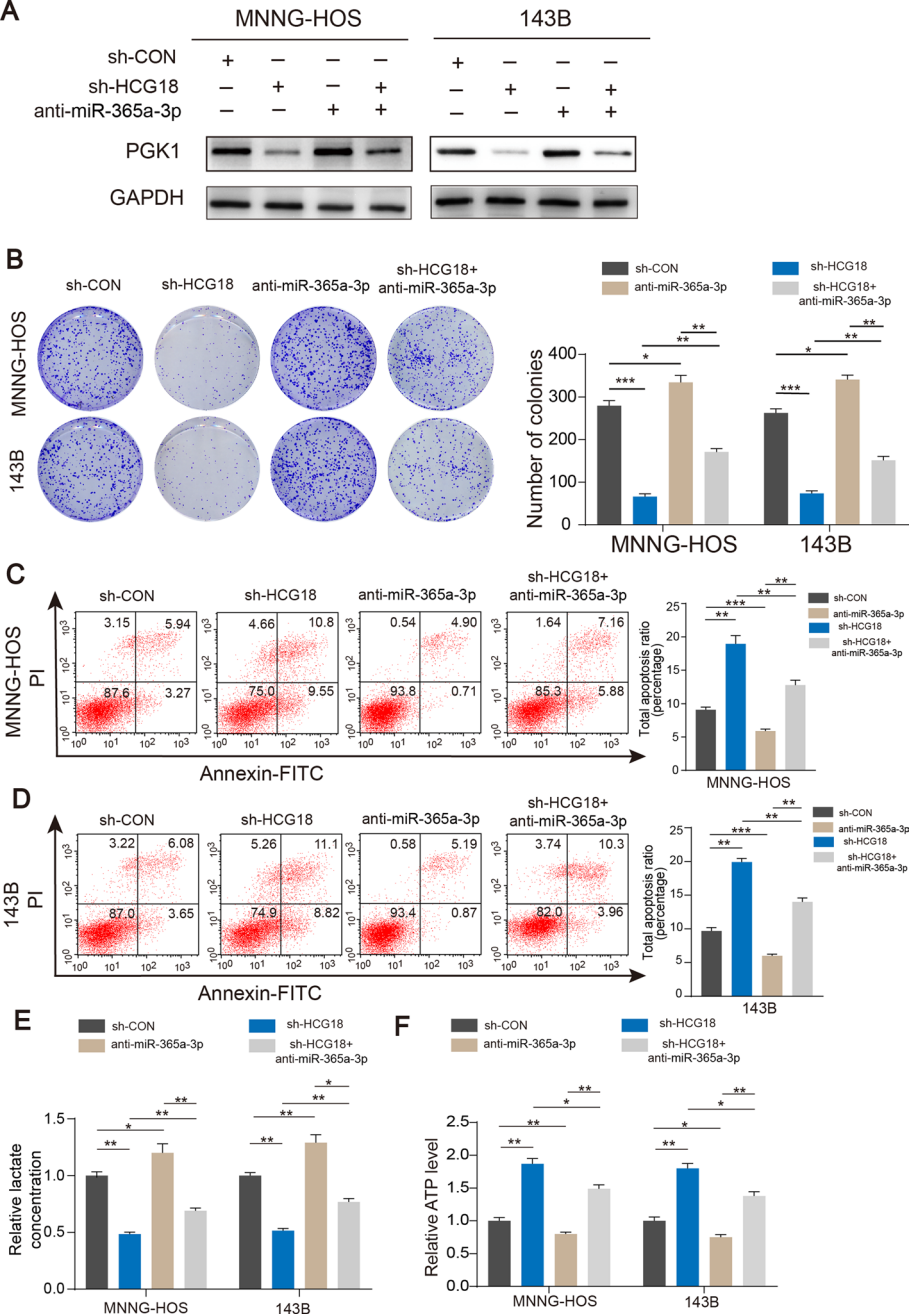
MiR-365a-3p inhibition reversed the inhibitory effect induced by knockdown of HCG18 in OS cells.** A** Western blot showed the PGK1 expression of OS cells transfected with miR-365a-3p inhibitor, sh-HCG18, or negative control. Colony formation (**B**), and apoptosis (**C** and **D**) assays confirmed that knockdown of miR-365a-3p partly reversed the suppressive effects of HCG18 knockdown on OS proliferation. Knockdown of miR-365a-3p in wild-type OS cells (MNNG-HOS and 143B) also improved OS proliferation. **E** and **F**, Altered levels of lactate concentration and generative ATP in different groups (sh-Control, sh-HCG18, sh-HCG18 + anti- miR-365a-3p and anti- miR-365a-3p) was measured. * represents *P* < 0.05, ** represents *P* < 0.01, *** represents *P* < 0.001. All experiments were repeated three times

In order to determine whether HCG18 affected cell growth through miR-365a-3p, OS cells were transfected with sh-HCG18 plus miR-365a-3p inhibitors as a rescue experiment. Western blotting showed that HCG18 knockdown greatly reduced PGK1 protein expression, while miR-365a-3p knockdown partly rescued PGK1 expression in OS cells (Fig. 6A). As depicted in Fig. 6B–D, miR-365a-3p knockdown reduced the suppressive effect of HCG18 knockdown on OS proliferation. Moreover, miR-365a-3p inhibition also restored the aerobic glycolysis that was inhibited by HCG18 knockdown in OS cells (Fig. 6E and F).

### HCG18 knockdown inhibited tumor growth in vivo

**Fig. 7:**
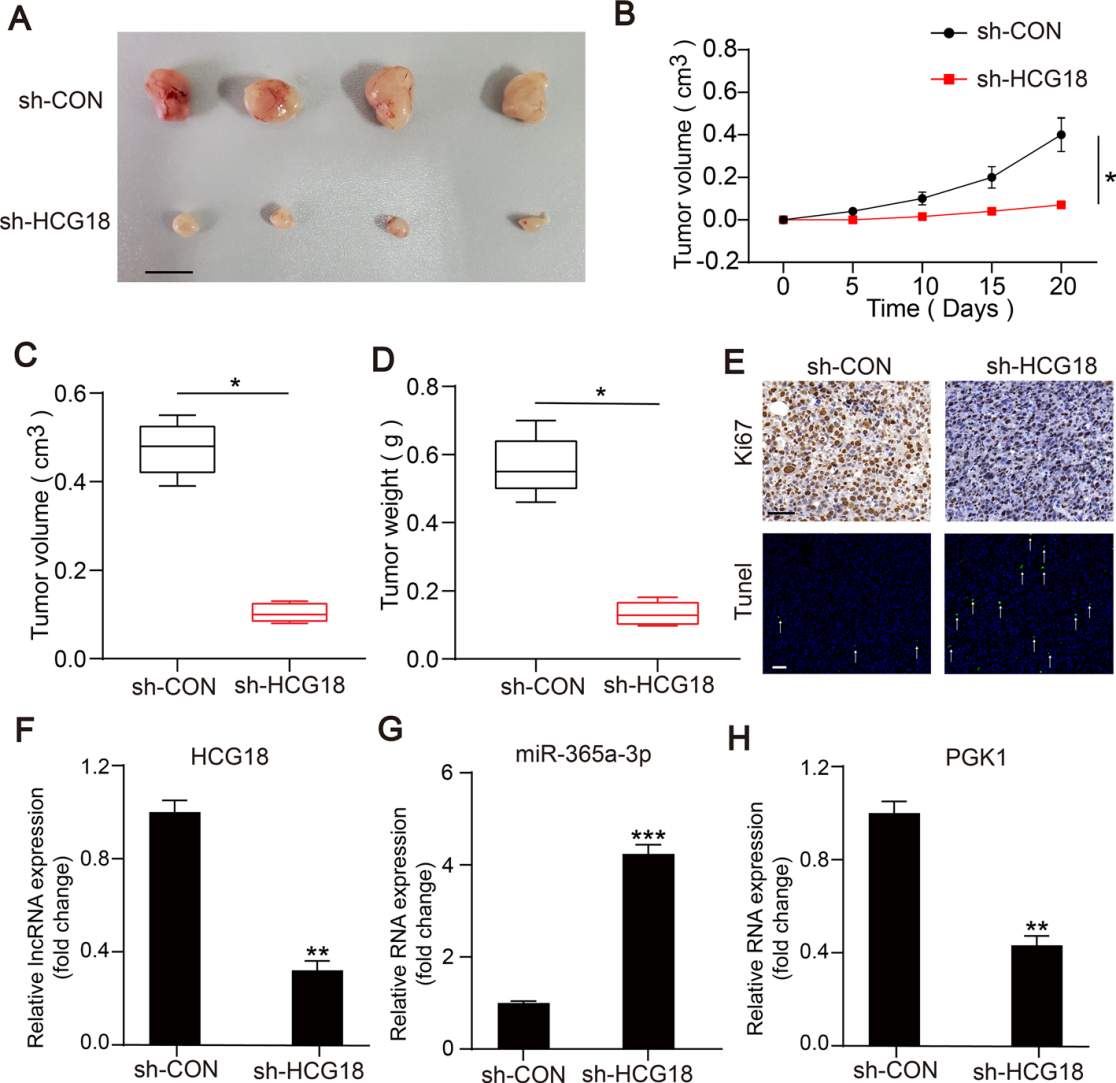
Knockdown of HCG18 inhibited OS cell growth in vivo.** A** An image of tumor tissues is presented. Scale bars = 1 cm. **B-D** Tumor growth curve, tumor volume and weight in sh-HCG18 group and sh-NC group were measured. **E** Expression of Ki67 and the rate of apoptosis in the xenograft tumors from the sh-HCG18 and sh-Control mice were monitored. A TUNEL positive cell is indicated (arrow). **F–H** Expression of HCG18, miR-365a-3p and PGK1 was monitored in xenograft tumors from sh-HCG18 group and sh-NC group. * represents *P* < 0.05, ** represents *P* < 0.01, *** represents *P* < 0.001. All experiments were repeated three times

An in vivo experiment was performed to explore the function of HCG18 in tumorigenesis. Stable HCG18-knockdown or control MNNG-HOS cells were subcutaneously injected into nude mice. We confirmed that tumors derived from the stable HCG18-knockdown group grow more slowly than those derived from the control group. Consistently, the tumor volume and weight in stable HCG18-knockdown were lower than those in the control group (Fig. 7C and D). Staining of tumor sections showed that Ki67 expression decreased and apoptosis rate increased in the HCG18-knockdown group compared with the control group (Fig. 7E). On the basis of the above results, we found that HCG18 act as an oncogenic factor in OS.

## Discussion

The lncRNA HCG18 has been reported to participate in cell growth and metastasis in various cancers. In gastric cancer, HNF1A increased HCG18 expression and HCG18 promoted cancer progression through the miR-152-3p/DNAJB12 axis [41]. Zou et al. demonstrated that HCG18 served as a ceRNA that promotes hepatocellular carcinoma proliferation via the miR-214-3p/CENPM axis [16]. In lung adenocarcinoma, HCG18 served as an oncogene and enhanced tumor progression by targeting the miR-34a-5p/HMMR axis [42]. In this study, our findings showed that HCG18 acted as an oncogene and played an essential role in OS cell growth.

MiRNAs are defined as small endogenous molecules without protein-coding ability and can have an important role in cancer progression [43, 44]. For example, miR-1297 was reported to suppress OS proliferation by regulating PFKFB2 [45]. MiR-365a-3p is also a critical participant in regulating cancer growth. Yang et al. reported that miR-365a-3p suppressed the Warburg effect and gastric cancer proliferation by regulating the HELLS/GLUT1 axis [38]. In colorectal cancer, miR-365a-3p reduced the cell growth and metastasis by regulating ADAM10/JAK/STAT signaling [39]. The current study confirmed that miR-34a-5p functioned as a tumor suppressor in pancreatic cancer by inhibiting c-Rel-mediated NF-κB signaling [46]. However, the role of miR-365a-3p in OS remains unclear. This study demonstrated that miR-365a-3p was downregulated in OS cell lines and miR-365a-3p overexpression suppressed OS cell proliferation.

Increased aerobic glycolysis was shown to accelerate cancer progression and cell growth, particularly by providing a large amount of intermediates for many biological signaling pathways and adapting to hypoxic condition [47]. Therefore, the reduction of aerobic glycolysis has become a therapeutic target in cancer treatment. However, how lncRNA regulates aerobic glycolysis in OS cells has not been studied in detail. In this study, we found that HCG18 is a novel promoter of aerobic glycolysis in OS by sponging miR-365a-3p, thereby upregulating expression of PGK1, which is a critical glycolytic enzyme.

## Conclusions

Our study demonstrated that HCG18 acted an oncogene and its expression was upregulated in OS. The results of our study demonstrated a critical role for HCG18 in the regulation of aerobic glycolysis by sponging miR-365a-3p to elevate PGK1 expression in OS cells. Therefore, HCG18 may be a potential therapeutic target in OS treatment.

## Supplementary Information

**Additional file 1: Figure S1.** HCG18 regulated expression of PGK1.

**Additional file 2: Figure S2.** MiR-365a-3p inhibit OS growth.

## Data Availability

The data supporting the conclusions of this article are available from the corresponding author on reasonable request.

## Abbreviations

OS: Osteosarcoma
ncRNA: Non-coding RNA
lncRNAs: Long non-coding RNAs
RT-qPCR: RNA extraction and real-time quantitative PCR
ceRNA: Competing endogenous RNA
ECAR: Extracellular acidification rate
OCR: Oxygen consumption rate
WT: Wild-type
Mut: Mutated
RIP: RNA immunoprecipitation
miRNAs: MicroRNAs
