# Supplementary figures and images for: LncRNA HCG18 promotes osteosarcoma growth by enhanced aerobic glycolysis via the miR-365a-3p/PGK1 axis
Source: Cell Mol Biol Lett. 2022 Jan 6;27:5. doi: 10.1186/s11658-021-00304-6 (PMC8903679; doi:10.1186/s11658-021-00304-6)

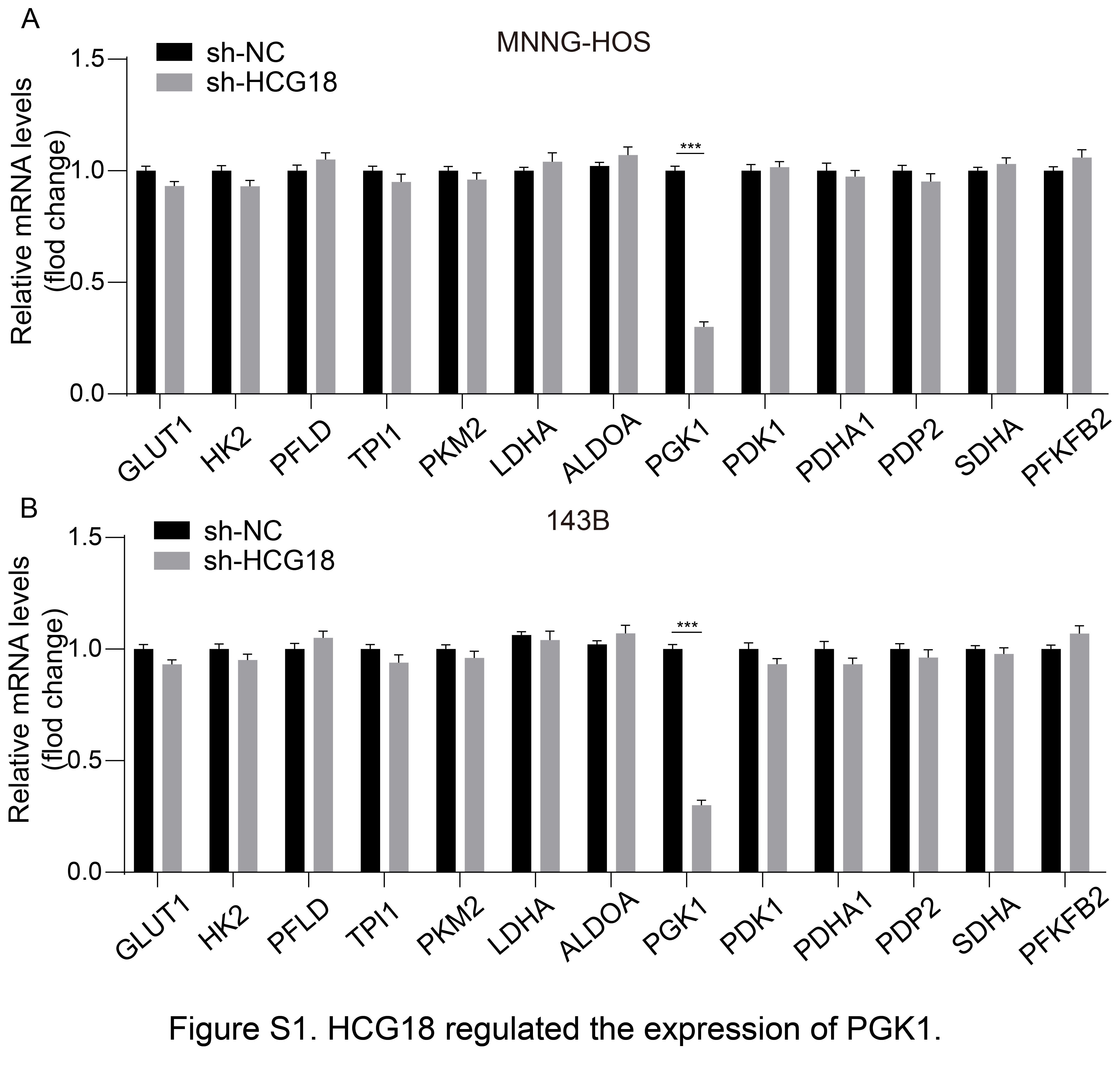

Supplement: Supplementary file 1 — Additional file 1: Figure S1. HCG18 regulated expression of PGK1. [file 11658_2021_304_MOESM1_ESM.tif]

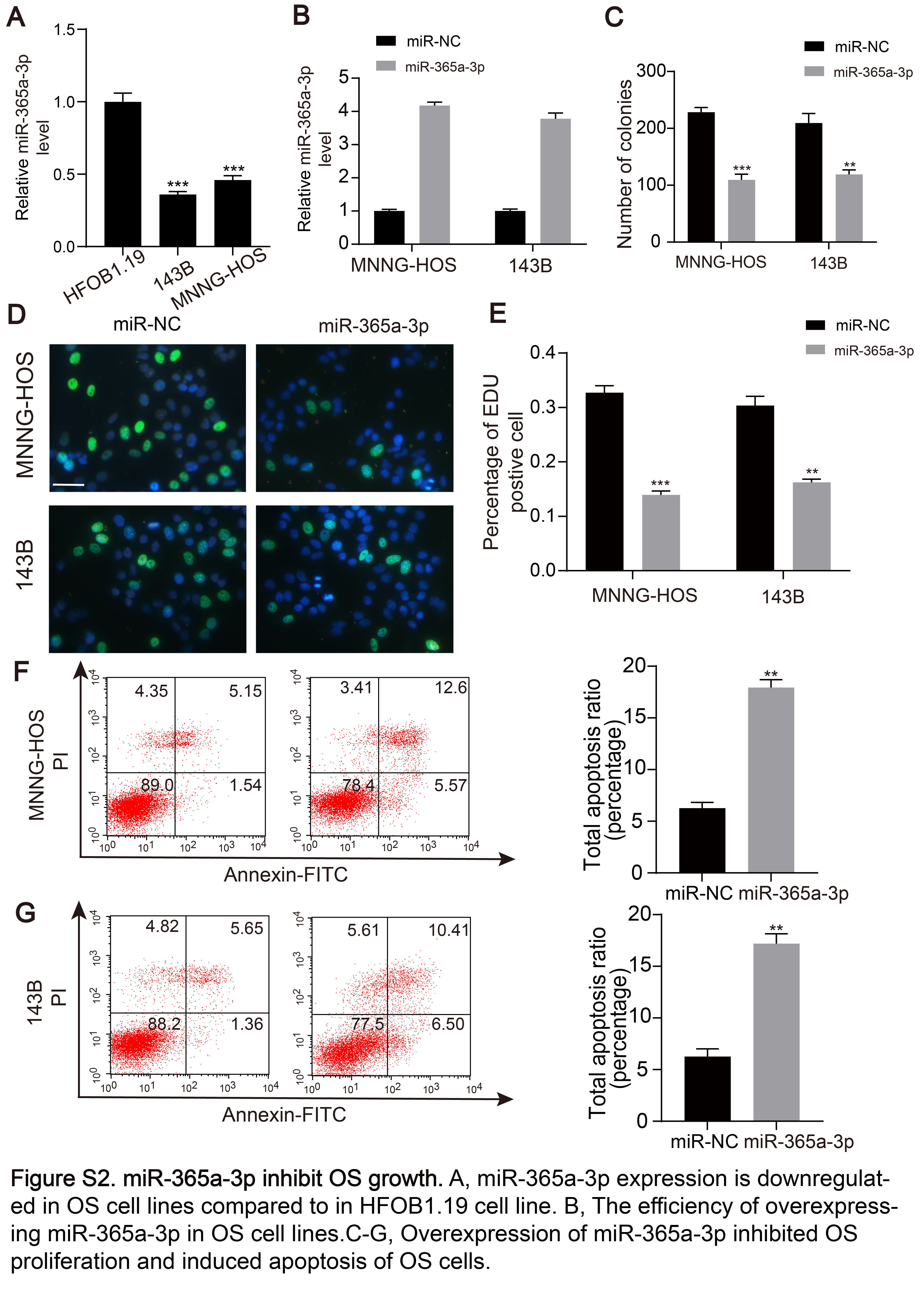

Supplement: Supplementary file 2 — Additional file 2: Figure S2. MiR-365a-3p inhibit OS growth. [file 11658_2021_304_MOESM2_ESM.tif]
